# Transient Catalytic Reaction Analysis Through Signal Defragmentation

**DOI:** 10.3390/e28040459

**Published:** 2026-04-17

**Authors:** Stephen Kristy, Shengguang Wang, Jason P. Malizia

**Affiliations:** Catalysis and Transient Kinetics Group, Idaho National Laboratory, 1955 Fremont Avenue, Idaho Falls, ID 83415, USA; stephen.kristy@inl.gov (S.K.); shengguang.wang@inl.gov (S.W.)

**Keywords:** mass spectrometry, temporal analysis of products, propane dehydrogenation, catalysis, defragmentation

## Abstract

The Temporal Analysis of Products (TAP) pulse response technique provides valuable insights into catalytic function and reaction kinetics. However, complex fragmentation patterns in the TAP mass spectrometry signals can complicate precise quantification, particularly when analyzing transient gas flux data typical of TAP experiments. This work demonstrates a standard defragmentation method that deconvolves transient TAP signals while maintaining the temporal resolution of the experiment. First, the integrals of calibration gas fluxes are used to determine the fingerprint fragmentation pattern and construct a fragmentation matrix. This matrix is then used to defragment experimental flux data at each recorded time point via a non-negative least squares regression. The effectiveness of this method is demonstrated using virtual data and control experiments with a TAP reactor system. The defragmentation is then applied to the more complex propane dehydrogenation reaction on a chromia/alumina catalyst, which can contain up to ten significant gas species in the reactor outlet. Initial propane pulsing reveals an induction period during which propane is fully oxidized to CO_2,_ followed by partial reduction to CO. Afterwards, there is a transition in chemistries towards coking and propylene production. Our example illustrates a practical method for the accurate determination of the time-dependent reactant/product concentrations and rates for a thorough analysis of the propane dehydrogenation kinetics. This approach can be broadly applied to any transient mass spectrometry experiment for a better understanding of catalyst-reaction dynamics.

## 1. Introduction

An in-depth understanding of catalysts and their interactions with reagent and product molecules enables advanced catalyst design and implementation. Mass spectrometry (MS) is a commonly used tool for detecting gas-phase species in catalyzed reactions operated under steady-state conditions [1,2,3,4,5]. However, the study of catalytic reactions under transient conditions offers deeper insights into catalytic function. For example, transient analysis can reveal how catalyst morphology changes over the course of light-off style activations [6] or how to improve the performance of desired chemistries through composition transitions [7]. Such discoveries are only possible through quantitative analysis of gas-phase species.

The Temporal Analysis of Products (TAP) pulse response methodology is a principal technique for understanding the transient kinetics of complex catalytic reactions. Typically, experiments will evaluate a range of mass to charge ratios (*m*/*z*) of interest, repeating scans to increase the experimental signal-to-noise ratio. TAP distinctively utilizes mass spectrometry: by sacrificing the range of masses to be monitored, the time-resolved pulse response of a small selection of masses (e.g., reactant or products) is achieved. Generally, plausible reaction products are known a priori, and the method will cycle through a set of mass-to-charge ratios (ca. 7–12 or more) to measure the time-dependent response at 1–10 kHz. This provides information on the function, utilization, and limitations of chemical reactions involving catalysts [8,9].

TAP enables the extraction of fundamental kinetic parameters from MS measurements by eliminating complicating effects such as non-isothermal conditions, convective flow, and gas-phase interactions, which are typical of steady-state flow reactors. In the TAP regime, gas–gas interactions are assumed to be negligible and transport is assumed to be governed by Knudsen diffusion, simplifying the extraction of intrinsic kinetic parameters. Such effects—like convective flow or intermolecular interactions—can obscure accurate kinetic measurements in traditional flow reactors.

Chemical manufacturers and catalyst researchers are increasingly interested in dehydrogenation reactions for producing light olefins, as demand has outpaced supply for decades [10]. Propane dehydrogenation (PDH) is a well-studied example of using an abundant resource as an alternative feedstock for propylene production [11,12]. However, the propane dehydrogenation reaction (PDH) presents a significant challenge for time-resolved transient catalytic reaction studies. Specifically, propane and all potential products from dehydrogenation or side reactions (e.g., cracking) fragment into multiple ions in the MS. This causes a convoluted time-varying MS signal with overlapping contributions at a given *m*/*z*, complicating quantitative interpretation of yields, conversions, and kinetics. Standard TAP data treatment typically includes de-fragmentation or similar approaches to separate overlapping fragment signals [13,14]. However, applying these procedures while preserving the full temporal resolution of transient MS traces remains a practical challenge in many datasets.

In this work, we apply a calibrated fingerprint–based defragmentation procedure to transient TAP MS measurements. The approach uses experimentally determined fragmentation patterns to separate overlapping signals in the raw MS data while maintaining the original time resolution, enabling more reliable quantification under fast transient conditions. Additionally, the incorporation of the NNLS algorithm ensures accurate signal deconvolution by preventing the formation of unphysical negative signals, enhancing the accuracy and reliability of the extracted data. This work, along with the provided Supporting Information, serves as a step-by-step guide for users seeking to implement these techniques, offering validation through virtual, control, and experimental datasets to ensure accurate and reliable application in transient TAP-MS analysis.

Virtual data and control experiments using the TAP reactor demonstrate the method’s functionality and effectiveness. After establishing proof of concept, the method is applied to study the evolution of the PDH reaction on a chromia/alumina catalyst (CrO_x_/Al_2_O_3_) following oxygen treatment. The method enables analysis of how the catalyst–chemistry relationship evolves from propane total oxidation to partial oxidation and ultimately to dehydrogenation. It is also found that a period of rapid coking occurs during the onset of propane partial oxidation. These “regimes” of activity are only identifiable from the precise titration experiments, quantitative mass balance analysis, and kinetic analysis. The changes in selectivity, combined with discrete changes in the kinetics of propane reaction rates, are evaluated in the form of rate-concentration petals. This kinetic analysis specifically highlights the independence of surface coverage during complete oxidation, followed by a transition to a coverage-influenced reaction as carbon accumulates and dehydrogenation takes over. This defragmentation method can be broadly applied to transient-based MS systems for deconvoluting complex chemical reactions, enabling a deeper understanding of catalyst–reaction systems.

## 2. Materials and Methods

The TAP instrument (Idaho National Lab, Idaho Falls, ID, USA) consists of a quartz micro-reactor. During calibration experiments, it is packed with acid-washed, rinsed, and calcined quartz (~200–300 µm diameter). The reactor sits directly over an MS ionizer housed in a vacuum chamber pumped by turbomolecular and diffusion pumps. During experiments, one or more pulse valves inject nanomolar amounts of gas into the evacuated (~10^−8^ torr) reactor, with the outlet gas flux recorded by the MS. Further details and schematics of the TAP instrument can be found in other publications [9,15].

A RGA200 mass spectrometer (Stanford Research Systems, Sunnyvale, CA, USA) operated at 1 amp (A) ionizer current and 1400 volts (V) channel electron multiplier voltage was used for gas detection. The gas flux data is collected by parking the MS detection at a single *m*/*z* for a single pulse, then cycling the *m*/*z* through each gas of interest’s main fragmentation peaks for subsequent pulses. The signal from the MS is amplified with a transimpedance amplifier with a gain of 10^−8^ A/V. A pulse collection time of 6.1 s is used with 0.5 s of dead time between each pulse. In all experiments, the reactant gas is mixed with 50 molar % of inert gas (helium (He) or argon (Ar)).

During PDH reactions, 20 mg of CrO_x_/Al_2_O_3_ (Clariant Corporation, Munich, Germany [16,17]) catalyst is loaded into the center of the reactor, surrounded by quartz, the TAP thin zone configuration [18]. The catalyst is pretreated in a 50:50 O_2_:Ar flow (40 standard cubic centimeters per minute (sccm)) for 30 min at 560 °C (ramp at 15 °C/min from ambient), followed by propane pulsing until conversion breakthrough occurs (~26 h). The small pulses deliver 6.7 nanomoles (nmol) of reactant gas per pulse and are in the Knudsen diffusion regime [19]. The data here are examined by slicing the experiment into periods of 500 pulses—cycling through *m*/*z* = 2, 15, 18, 26, 28, 29, 30, 41, 44, plus either 4 or 40 depending on whether He or Ar is used as the inert in pulsing—then averaging the 50 pulses per *m*/*z* together. The averaging is done after defragmentation calculations are collected for all data. Averaging is done only to provide ocular clarity to the reader. Unaveraged data are found in Figure S8.

The thin zone packing and operating temperatures prevent either escape or volatilization of catalyst materials into the MS chamber in this experimental setup.

Before defragmentation and analysis, the TAP data is preprocessed with gain calibration and background subtraction. Additional details regarding preprocessing and experimental procedures are found in the SI and figure captions.

The CrO_x_/Al_2_O_3_ catalyst (13.2 wt% Cr) was supplied by Clariant Corporation (Clariant Corporation, Munich, Germany).

## 3. Results

### 3.1. Proof of Concept

In TAP experiments, nanomolar amounts of gas are injected into a packed reactor. The resultant flux from this injection is measured at the reactor exit with an MS. The general procedure for deconvolving overlapping *m*/*z* caused by gas fragmentation in TAP is (1) Collection of the fragmentation pattern for possible reagents/products over an inert packed reactor at identical reaction conditions as the reaction of interest (temperature, pressure, pulse size, MS settings, etc.). The zeroth moment (integral of the flux response) is used to construct a fragmentation matrix from calibrations. Notably, National Institute of Standards and Technology (NIST) fragmentation patterns should not be used to construct the fragmentation matrix, as gas fragmentation varies from MS to MS and with the reactor/MS chamber conditions. Due to variations in ionization efficiency between different mass spectrometers and the sensitivity of fragmentation patterns to factors such as chamber pressure, gas temperature, and filament degradation over time, NIST fragmentation data may not accurately reflect the behavior of specific MS systems. Instead, fragmentation patterns should be experimentally determined under the exact operating conditions of the TAP-MS to ensure reliability and reproducibility. (2) During the reaction over the catalyst, apply the calibration fragmentation matrix to deconvolute the flux response point by point via a non-negative least squares (NNLS) regression. Generally, the NNLS is used to solve Ax = b for x, where A is the fragmentation matrix, b the fragmented (raw *m*/*z*, experimental) data, and x the unknown gas concentration. A discussion, walkthrough, and mathematical equations for these steps are provided in detail in the Supporting Information (SI).

**Figure 1:**
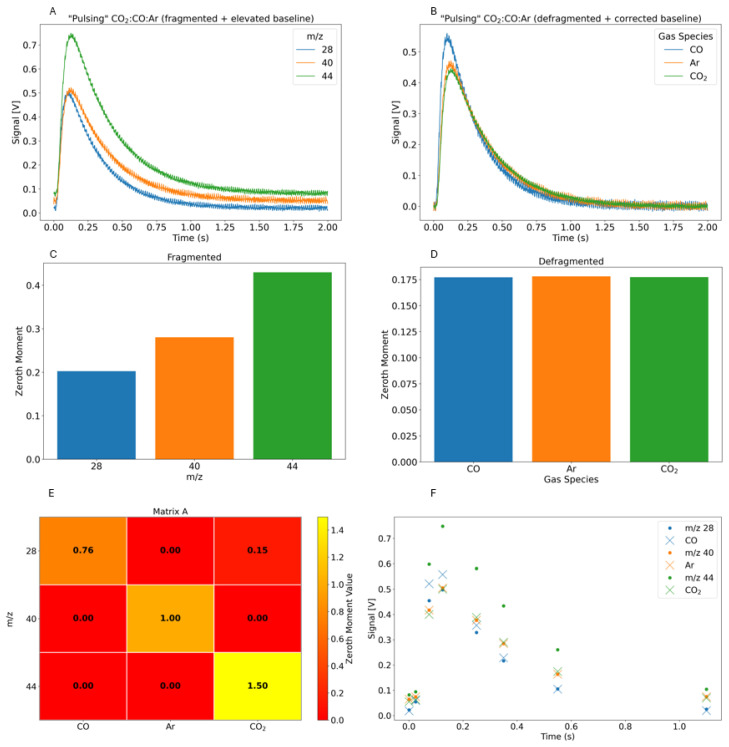
VTAP pulsing CO, CO_2_, and Ar in equal amounts and defragmentation results. (**A**) Simulated flux response curves. (**B**) Defragmented/baseline corrected flux curves (**C**) Simulated zeroth moment from VTAP. (**D**) Baseline corrected and defragmented zeroth moment from B. (**E**) Defragmentation matrix. (**F**) Eight data slices in time to illustrate the data before (circle) and after (X) defragmentation.

CO and CO_2_ offer a useful example to display the concept and effectiveness of the defragmentation method employed here, since these gases have an overlapping fragment at *m*/*z* 28. In Figure 1A, the virtual TAP reactor model (VTAP) [20] was used to simulate an experiment where CO, CO_2_, and Ar are pulsed in tandem. VTAP is a simulation framework connecting the exit flux with the gas concentration profile in the TAP reactor and can mimic the fragmentation process to generate simulated datasets for method validation. The VTAP simulation pulses in equal molar amounts of CO, CO_2_, and Ar. The exit gas flux is converted to fragmented signals by multiplying with an arbitrary scaling factor that mimics differences in ionization efficiency (0.76 for CO, 1 for Ar, 1.5 for CO_2_), then adding the contribution of CO_2_ to the *m*/*z* 28 signal (10% of the *m*/*z* 44 signal).

A random baseline and experimentally collected background noise are added to the simulated flux signals to more closely resemble a real experiment (Figure 1A).

A fragmentation matrix (Figure 1E) is constructed using VTAP simulations mimicking a typical TAP calibration experiment, where 50:50 mixtures of CO_2_:Ar and CO:Ar are pulsed (SI Figure S1). This matrix is then applied point by point to the raw flux (*m*/*z*) signal to solve the unknown gas concentration via NNLS. Figure 1F displays 8 data slices in time to illustrate the data before (circle) and after (X) defragmentation. All 2000 slices in time are put through the NNLS and are reconstructed into the final data illustrated in Figure 1B,D. By comparing the zeroth moment of the defragmented flux (Figure 1D) to the input VTAP parameters (equal parts CO, CO_2_, Ar), we observe that this method reproduces the original gas concentrations with 99% accuracy.

### 3.2. Control Experiment

**Figure 2:**
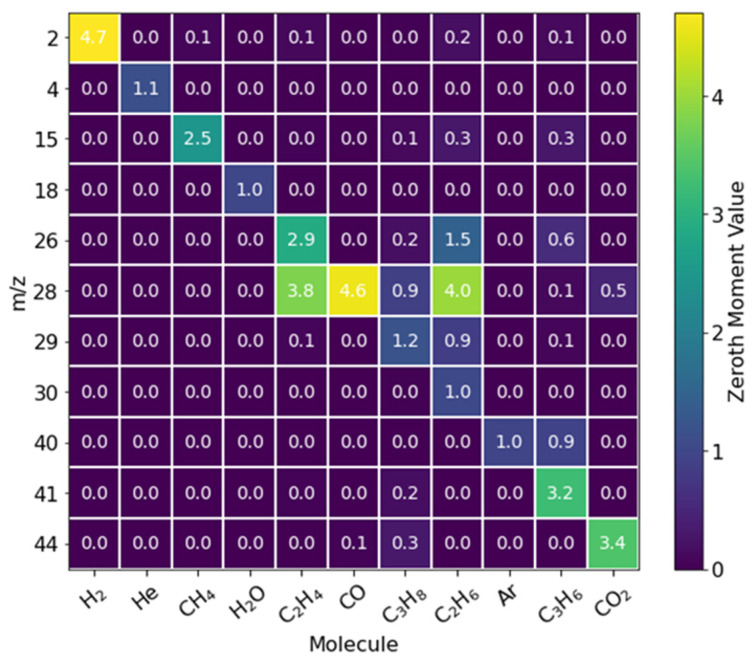
A fragmentation matrix constructed from calibration data for a PDH reaction. The colors represent the zeroth moment values acquired during calibration.

To ensure the defragmentation process can correctly deconvolute complex-overlapping fragmentation patterns, we first provided a simulated CO:CO_2_:Ar co-pulse using the virtual TAP model (Figure 1). We now pivot to a more complex example where propane and propylene are pulsed in tandem to illustrate that the overlapping *m*/*z* signals can be fully deconvoluted in the real TAP experiment via the standard methodology. Here, 50:50 mixtures of propane:Ar and propylene:He are pulsed through two separate pulse valves into an inert packed reactor at 560 °C. The size of the two pulses is ~2:1, respectively. The mass spectrometer cycles through 12 different *m*/*z* values to monitor masses associated with possible side products of a PDH reaction (e.g., CH_4_, CO_2_, etc.). The resulting data are then deconvoluted using a fragmentation matrix constructed from calibration measurements (Figure 2). Since the only gases introduced to the system are propane, propylene, and their respective inert counterparts, successful defragmentation will show contributions from only those four species.

**Figure 3:**
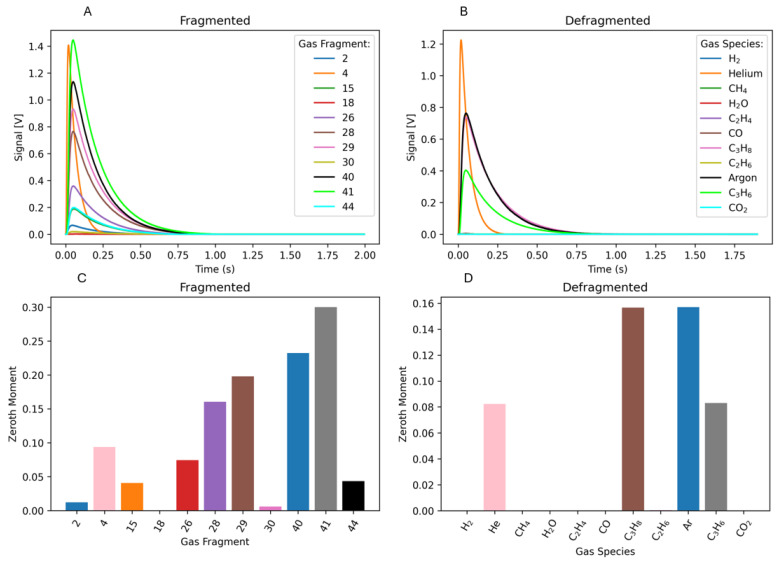
Pulsing propane:Ar and propylene:He over an inert packed TAP reactor at 560 °C. (**A**) Background subtracted, raw *m*/*z* signal experimental flux data and (**B**) defragmented flux data representing gas species. (**C**) Zeroth moment of the raw *m*/*z* experimental data and (**D**) zeroth moment of the defragmented flux gas species.

Figure 3 displays the fragmented (A) (raw *m*/*z* signals) and defragmented (B) gas species flux for this experiment, in addition to the fragmented (C) and defragmented (D) zeroth moments. There are many *m*/*z* fragments (Figure 3A,C) being picked up that are not representative of what was pulsed over the inert reactor. For example, *m*/*z* 28 is the primary (largest) fragment for gas species CO, ethane, and ethylene. This flux response shows up when pulsing the propane:Ar and propylene:He combination, which is a misrepresentation of the actual gas composition. Following defragmentation, the gas concentrations consist entirely of propane, propylene, Ar, and He (Figure 3B,D). As expected from the different pulse sizes, we observe a ~2:1 ratio of propane to propylene. In-depth details of this calculation, like those found in Figure 1E,F, are found in the SI Figures S3 and S4.

### 3.3. Catalyst Experiment

**Figure 4:**
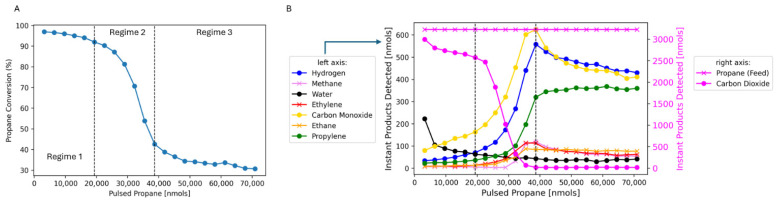
Propane conversion versus propane pulsed into the TAP (**A**). Results are for 20 mg of oxidized CrO_x_/Al_2_O_3_ at 560 °C. Each data point is an average of 500 Knudsen-diffusion-sized TAP pulses. (**B**) The right axis dictates CO_2_ and Propane feed quantities, left axis indicates all other yields. The conversion of moments to moles and further details on yield and conversion calculations are discussed in the SI.

**Figure 5:**
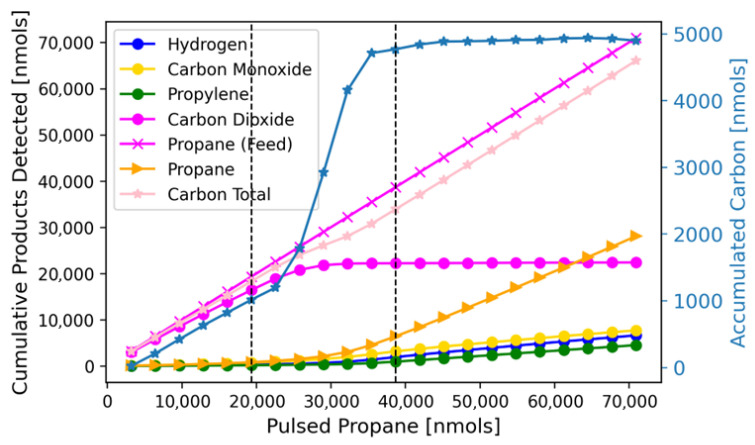
Cumulative molar quantities exiting the TAP reactor are calculated via summation of all carbon products detected and known feed. The right axis (light blue) indicates the carbon accumulation as the difference in feed and total carbon exiting. Results are for 20 mg of oxidized CrO_x_/Al_2_O_3_ at 560 °C.

**Figure 6:**
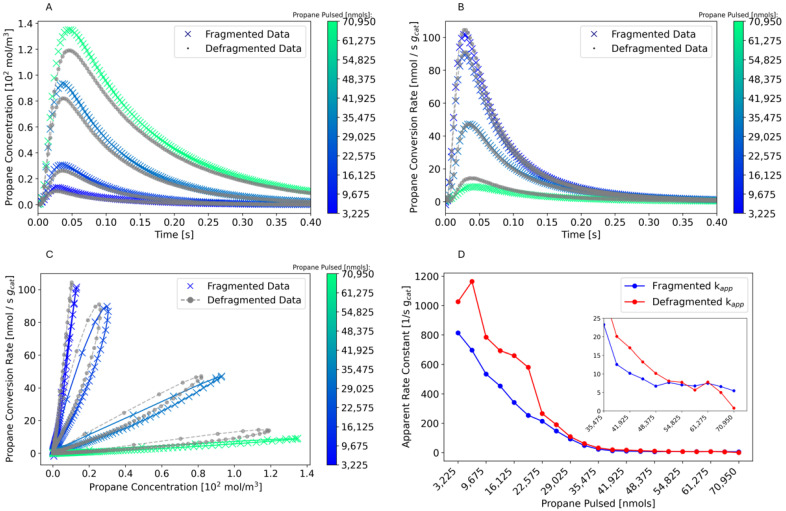
Comparison of propane reaction rate and concentration before and after defragmentation during propane pulsing (PDH). In (**A**–**C**), the yellow-purple colored data represents the curves before defragmentation based on the raw *m*/*z* = 29 flux data, gray data represents the curves after defragmentation. Four data series are shown in (**A**–**C**), representing propane exposure of 3 × 10^3^, 2 × 10^4^, 3 × 10^4^, and 7 × 10^4^ nmols, respectively. (**A**) illustrates the concentration-time response, (**B**) illustrates the rate-time response, and (**C**) illustrates the rate-concentration petals. (**D**) displays the estimated reaction rate constant before and after defragmentation. Results are for 20 mg of oxidized chromia/alumina at 560 °C.

**Figure 7:**
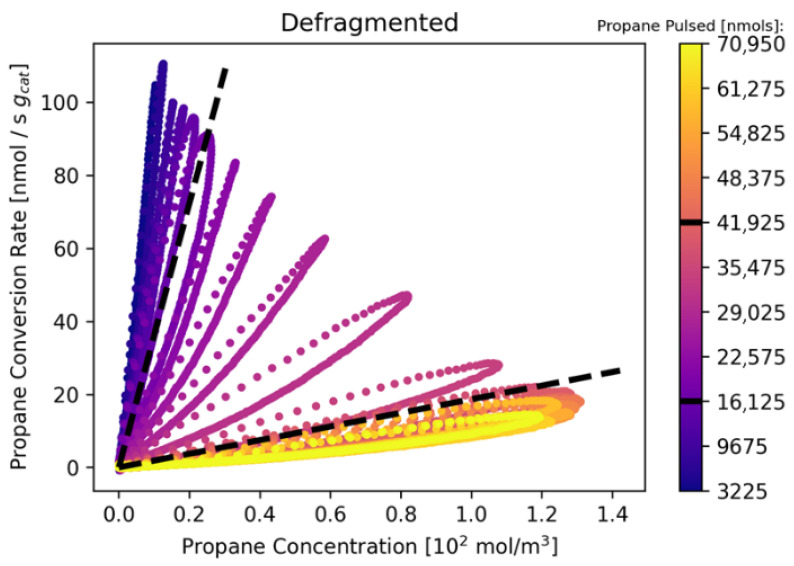
Propane Reaction rate and Concentration petals calculated with the G procedure. The amount of nmols of propane pulsed follows the color scheme purple to yellow. All y units = Rate [nmol/s g_cat_]. All x units = Concentration [10^2^ mol/m^3^]. Black dashed lines represent reaction regimes (see text).

In the case of TAP experiments on real catalysts, transient MS data can be utilized to acquire an understanding of chemical reaction pathways through precise quantification of reaction products and calculation of kinetic parameters. We illustrate this through the analysis of a TAP experiment monitoring the PDH reaction on an oxidized CrO_x_/Al_2_O_3_ catalyst at 560 °C. Defragmented flux signals are provided in the SI Figure S9. Those fluxes are used to calculate conversion and product yields from their zeroth moment (Figure 4), cumulative product yield (Figure 5), and rate-concentration data (Figure 6 and Figure 7). Notably, the data in the main text have been prepared by averaging every 500 pulses; the unaveraged data are provided in the SI (Figure S8).

The conversion, yield, and mass balance data reveal three distinct regimes during PDH, marked with vertical lines in the figures. In regime 1, there is nearly complete propane conversion, where CO_2_ is the main product. This reaction does not produce H_2,_ although considerable water (uncalibrated) is observed. The water trend combined with CO_2_ yield, along with a lack of other products in significant quantities, indicates regime 1 to be the total oxidation of propane. While this ‘combustion’ regime is typically missed in traditional flow experiments, it has been observed in other pulse response studies on CrO_x_/Al_2_O_3_ [19,20].

In regime 2, propane conversion rapidly decreases while CO, H_2_, propylene, and other minor product yields increase. The significant CO yield indicates partial propane oxidation by the catalyst. Previous studies by our group, enabled by operando characterization of the Cr oxidation state, found this partial oxidation to be the reaction between propane and chromates to yield CO and Cr_2_O_3_ [20]. The rapid coking (carbon accumulation) observed here (Figure 5) suggests this redox reaction involves the deposition of carbon. The rise in propylene yield during regime 2 is due to the catalyst reduction, as Cr(3+) is known to be the PDH active site in this system [20,21,22]. Afterwards, the system enters regime 3, which is the PDH dominant regime.

One of the key capabilities of TAP is the ability to transform the exit gas flux into rates and concentrations at the catalyst zone. We illustrate this through the analysis of the propane concentration and reaction rate during PDH, extracted via the G procedure [21]. The Y procedure can also be utilized, and a comparison of the two techniques for this dataset is given in SI Figure S10. In Figure 6A,B, a comparison is made between the propane concentration and reaction rates at varying degrees of propane exposure, when the data is defragmented versus using the raw MS signal at *m*/*z* 29, propane’s main fragment. In all cases, the peak time, flux tail, and magnitude of the curves change over the entire duration of propane pulsing, depending on whether the data were defragmented. These changes are unique for each pulse cycle displayed over the course of the individual pulse response, and indicate the importance of defragmentation to extract accurate kinetic parameters. Figure 6C further illustrates the importance of defragmentation by comparing the rate-concentration (RC) petals for propane before and after defragmentation. RC petals are critical in TAP analysis as they enable the extraction of kinetic parameters such as rate constants and the number of active sites [22]. The rising edge of the petal is used to calculate the apparent rate constant for propane consumption. In all cases, the qualitative pattern of the petals changes with defragmentation, in addition to the extracted kinetic parameters, which changed by up to 100% after defragmentation.

### 3.4. Discussion

Figure 7 presents the defragmented RC petals for propane conversion, which serve as fingerprints of the reaction mechanism and catalyst surface state [20,22]. The petals evolve through the three regimes, each reflecting transient changes in catalyst surface chemistry. In regime 1 (dark purple petals), high propane conversion rates show initially linear rate-concentration (RC) relationships. Narrow petals suggest minimal influence of surface coverage, indicating that the combustion reaction initially proceeds with little dependence on catalyst composition. This could suggest the material (from pretreatment) oxygen is localized in/on the material surface and directly reacts with the gas phase propane. As propane pulses continue and carbon accumulates (~20,000 nmol propane exposure), petals widen, signaling the onset of surface coverage effects and the depletion of pre-treatment oxygen.

A subtle narrowing then dilation of petals marks the transition to regime 2, where combustion gives way to catalyst reduction. This is supported by the disappearance of CO_2_, increased CO formation, and early signs of carbon accumulation (Figure 4 and Figure 5). The temporary narrowing suggests a brief period where surface coverage has less impact, likely due to the loss of combustion-active species (oxygen). As propane continues to interact with the catalyst, petals widen again, indicating renewed surface sensitivity—possibly due to coking or chromia reduction [23,24]. This regime ends as propane conversion plateaus and CO and H_2_ yields rise, signaling the onset of PDH and side reactions.

After ~40,000 nmol of propane, RC petals stabilize, indicating a steady-state regime. Propylene, CO, and H_2_ become the dominant products (Figure 4B). Despite ongoing coking and catalyst reduction, the wide, consistent petals suggest sufficient active sites remain to sustain stable reaction trends. The continued CO formation implies partial oxidation persists, possibly due to limited access of the small propane pulses to bulk oxygen. Notably, this regime’s detail is obscured in the fragmented raw *m*/*z* data (Figure 6), highlighting the value of defragmentation.

## 4. Conclusions

This work demonstrated the application of moment-based TAP calibration data to defragment transient flux data with illustrative VTAP and control experiments. The procedure applied to the more complicated PDH TAP experiments demonstrates that transient MS data, when properly defragmented, can reveal intricate details about chemical reaction pathways and kinetic parameters. Throughout our analysis of the PDH reaction on a CrO_x_/Al_2_O_3_ catalyst, we have shown that accurate defragmentation of MS signals is crucial for determining the rates and concentrations of both reactants and products, thus ensuring precise and reliable data interpretation. The defragmentation process, in tandem with the G or Y procedure, transforms the flux at the reactor outlet to reflect the flux within the catalyst zone, enabling a clear depiction of reaction rates and yield trends. The results highlight the significant transitions in catalyst behavior across different regimes of propane pulsing, with distinct changes in product formation patterns and catalyst surface composition. These transitions, particularly from an oxygen-rich to a reduced surface, emphasize the necessity for standard defragmentation techniques.

## Data Availability

The defragmentation python code used in the study is openly available in Github at https://github.com/IdahoLabResearch/DEFRAG-MS (accessed on 8 March 2026). The raw data supporting the conclusions of this article will be made available by the authors on request.

## Supplementary Materials

The following supporting information can be downloaded at: https://www.mdpi.com/article/10.3390/e28040459/s1. References [25,26,27,28,29,30,31,32] are cited in the Supplementary Materials.

## Abbreviations

The following abbreviations are used in this manuscript:

TAP	Temporal Analysis of Products
MS	Mass Spectrometer(ry)
PDH	Propane Dehydrogenation
RGA	Residual Gas Analyzer
