# Supplementary material for: Transient Catalytic Reaction Analysis Through Signal Defragmentation
Source: Entropy (Basel). 2026 Apr 17;28(4):459. doi: 10.3390/e28040459 (PMC13114260; doi:10.3390/e28040459)
Supplement: Supplementary file 1 [file entropy-28-00459-s001.zip › entropy-4219983-supplementary.pdf]

Supporting Information

Defragmentation

Some reactions/gas mixtures have complex, overlapping fragmentation signals in MS, a process which is complicated further when aiming to achieve temporal clarity between gases, such as in a TAP experiment. To understand clearly what products and reactants are formed based on observed fragments during a reaction, calibrations are performed. These allow for an understanding of specific gas fragments in the specific mass spectrometer system. The National Institute of Science and Technology (NIST) offers example fragmentation patterns for most gases. [1] The NIST profiles are useful for understanding broadly which gas fragments may exist based on known reactants and expected products, but each MS\vacuum system is unique (filament current, multiplier voltage, pressure, etc.) and therefore these fragmentation patterns should only be used as a starting point.

A fragmentation matrix is constructed based on the fragmentation pattern observed by inputting equal molar amounts of all gases that will exist in the vacuum chamber for the specific measurement. To accomplish this for analysis of chemical reactions with an MS, 50:50 mixtures of gas:inert are delivered in equal molar amounts over an inert-packed reactor (e.g., acid-washed, rinsed, and calcined quartz, no chemical reaction) at the desired reaction temperature. The addition of the inert gas is vital as it provides a reference for the gas of interest's fragmentation and intensity. Therefore, the first step in defragmentation calibrations is knowing what gases will be present in the reaction of interest, i.e., reactants and products.

A detailed explanation of the construction of the fragmentation matrix via calibrations is given with required calculations in the form of equations, in the SI, Figure S5 (Defragmentation). Once the fragmentation patterns of reactants and expected products are recorded and the zeroth moment

is calculated for each individual fragment, a fragmentation matrix is constructed. In the case of TAP, the zeroth moment is the integral of the mass spectrometer signal over the time range of a pulse. This quantity can be calculated for any transient analysis using MS. [2] This matrix is therefore a constant fingerprint (based on the zeroth moments) of what the known fragmentation patterns are for the various gases that could be generated (or pulsed in) during a specific chemical reaction. In the case of TAP, these moment values describe each specific gas's behavior over the time it takes to record a pulse response, specific to each observed fragment. We note that the defragmentation calibration data needs to be collected around the same time and under the same reaction conditions as the target reaction.

The reason for the construction of this “fragmentation matrix” is the deconvolution of reaction, experimental data, where there are overlapping ion readings in the MS. This deconvolution can be done by considering calibrations and an experimental data set with the Ax=b linear algebraic form. In this case, (A) is the constant fragmentation matrix with known patterns from calibrations. Let (m) be the number of species and (n) be the total number of m/zs tracked. Then, A is an (m) by (n) matrix, and (x) is a vector of length (m), and (b) is a vector of length (n). In other words, (b) is the raw (fragmented) data we collect with the MS during the experiment, and (x) is the gas concentration. To solve this equation, we apply non-negative least squares regression (NNLS). NNLS is useful here and commonly employed when only positive values are realistic for the experiment at hand. [3] Equation (1) displays proper formatting when utilizing NNLS.

$$Ax = b \rightarrow x = NNLS(A, b) \quad (1)$$

Detailed justification of NNLS as the choice tool, along with simplistic examples of executing these procedures with simple data sets, is given below (NNLS execution).

Matrix construction: When calculating the values that make up the matrix, ensure the pulse response data are gain corrected, background subtracted, and that the data starts at the time when the pulse valve fires (for example, 0.1 s). Note, the background subtraction method should be consistent per amu/species from calibrations to experiments.

The matrix rows represent the fragments from the gas species you wish to track during the experiment. The columns are the observed fragments during defragmentation calibration of gas delivery over an inert reactor for each specific gas. The data in each column is therefore the zeroth moment for each fragment ion detected for each gas pulsed during calibrations. Now the moments are tabulated in your matrix, normalize the moment values by that of the inert, such that inert moments equal unity. Details on how to calculate zeroth moments are displayed in equations in Figure S 1. This step is required so that MS signals [V] can be converted to appropriate units (e.g., [nmol/g_{cat} s] in TAP applications) when rates and concentrations are calculated. Examples of such calculations are covered here [4]. This is a unitless multiplier, so at this point the flux units would remain [V]. During the calculation of rates and concentrations, discussed later in this text, a full conversion is made to [nmol/g_{cat} s] by multiplying the signal by the number of moles in a moment (single pulse). This step can be applied to flux without calculating rates or concentrations to present the flux in units of ([nmol/cm^2_{cat} s] or [nmol/g_{cat} s]) rather than a voltage. Details on how to calibrate and determine the moles per pulse for a unique pulse valve are reported elsewhere [4]. Following inert normalization, convert all inerts to the inert that will be delivered to the MS during the experiment (i.e., convert Helium moment to Argon moment). The matrix is now ready to be applied to an experimental dataset.

The details of this calculation/method are highlighted in Figure S1. The Figure represents the generation of a CO_2 column in a fragmentation matrix. The "raw" data is generated by VTAP [5].

VTAP is used to artificially add a fragmentation pattern to CO_2 (AMU 28), evaluate the baseline, and add noise to more closely resemble a real experiment. Note, this row would be transposed, such that the AMUs (fragments) make up the rows in the final table (as seen in the main text).

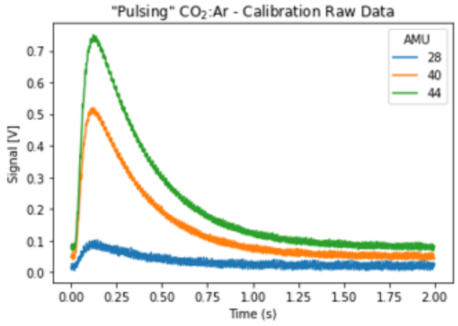

$$S_i^{bc}(t) = \left(S_i^{raw}(t) - B_i\right)$$

$$S_i^{gc}(t) = S_i^{bkg}(t) \cdot g_i = S_i$$

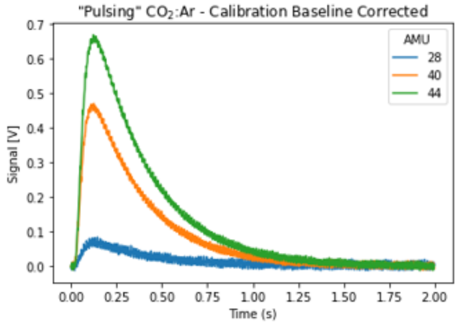

$$M_i = \int S_i(t)dt$$

AMU	28	40	44
M_i	0.027	0.178	0.266

$$M_i^{norm} = \frac{M_i}{M_{inert}}$$

AMU	28	40	44
M_i^{norm}	0.150	0.000	1.496

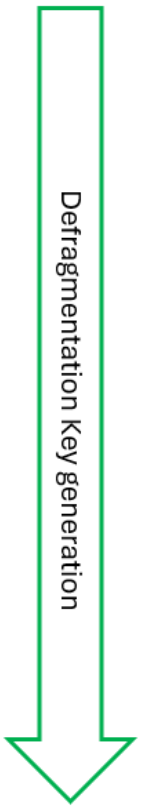

Figure S1– Generation of a single row (column) in a fragmentation matrix. The CO_2 is utilized, where artificial fragments and noise in the data are added. $S_i^{raw}(t)$= raw signal (fragments + gas species) for AMU I, B_i = baseline for AMU , gc = gain, corrected, bc = baseline corrected, g_i = gain correction factor, S_i = gain/baseline corrected MS signal, M_i = zeroth moment (fragments + gas species) of AMU I, t = time.

NNLS execution: To technically perform the NNLS, we must consider that the amount of data generated in TAP experiments is vast. A typical time scale for a single pulse is about 1-6 seconds at a 1000 Hz collection rate. The mass spectrometer, therefore, generates 6,000+ rows of data for each pulse. Depending on the number of pulses recorded, this can lead to a very large (b) vector. Therefore, to efficiently apply the NNLS to the dataset and matrix (A), each discrete row in time is individually solved, and the results are reconstructed into the full signal over time. Since the calibration matrix (A) is constant and not dependent on time, the relationship between (A) and each column of (b) is independent of other time points. This means that each time point can be solved as a separate NNLS problem without affecting the others. Even though (A) is constructed from moment values of the entire curve, these moment values are properties that capture the gas behavior over the time a flux response is recorded. (A) encodes the relative contributions of each fragment over the entire response period, making it applicable to individual time points. Therefore, the decomposition into individual time points does not alter these intrinsic properties. Each column of (b) is still being matched against the same (A), ensuring that the moment values are consistently applied to each time point. The linear superposition principle in linear algebra states that a linear combination of solutions to a linear system is also a solution. [6] By solving for each time point individually and then concatenating the results, we effectively leverage this principle. The overall solution (x) constructed from individual solutions remains valid and consistent with the linear relationship defined by (A). Finally, from a computational perspective, solving smaller, independent problems (one column at a time) can be more efficient and manageable than attempting to solve a large, complex system all at once. This approach allows for parallel processing and more straightforward debugging, without compromising the accuracy or integrity of the solution. The noise experienced with TAP pulse responses is white noise, indicating the

frequency of randomness is distributed equally, for both experiments and calibrations. This means the noise contains no patterns, is consistent from calibrations to experiments, and therefore should not violate the linear superposition principle. Note, we described how the moment values of (A) are valid when solving for the signal values of (x) from (b). These signals are then integrated to acquire defragmented zeroth moments. This means that the moment values of (A) generate the signal values in (x). To explain the validity of this approach, consider that for each time point in the NNLS iteration, every scanned AMU is subjected to the NNLS. For example, during the PDH, each time point solved in NNLS would have ten AMU values. The NNLS can use these ten AMU signals at each time point against the fragmentation matrix to deconvolute each point in time, which is then reconstructed into a full flux response. During PDH reactions, if propane is pulsed and no reaction occurs, the fragmentation pattern (ratio of AMUs collected) at each time point will match the propane row in the fragmentation matrix. For instance, the ratio of AMU 2:29 in the propane row of (A) will match the observed 2:29 ratio when only propane is present in the reactor. However, if a reaction occurs and hydrogen is formed, the AMU 2 ratio will not match the propane fragmentation row, but AMU 29 will not be affected by hydrogen. Therefore, the NNLS will see that the AMU 2:29 ratio does not match, but the propane value remains consistent with (A). This allows the NNLS to determine what fraction of AMU 2 is from propane based on the value at AMU 29, attributing the remainder of AMU 2 to hydrogen. Of course, during the PDH reaction, the ion fragmentation patterns are more complex than this example.

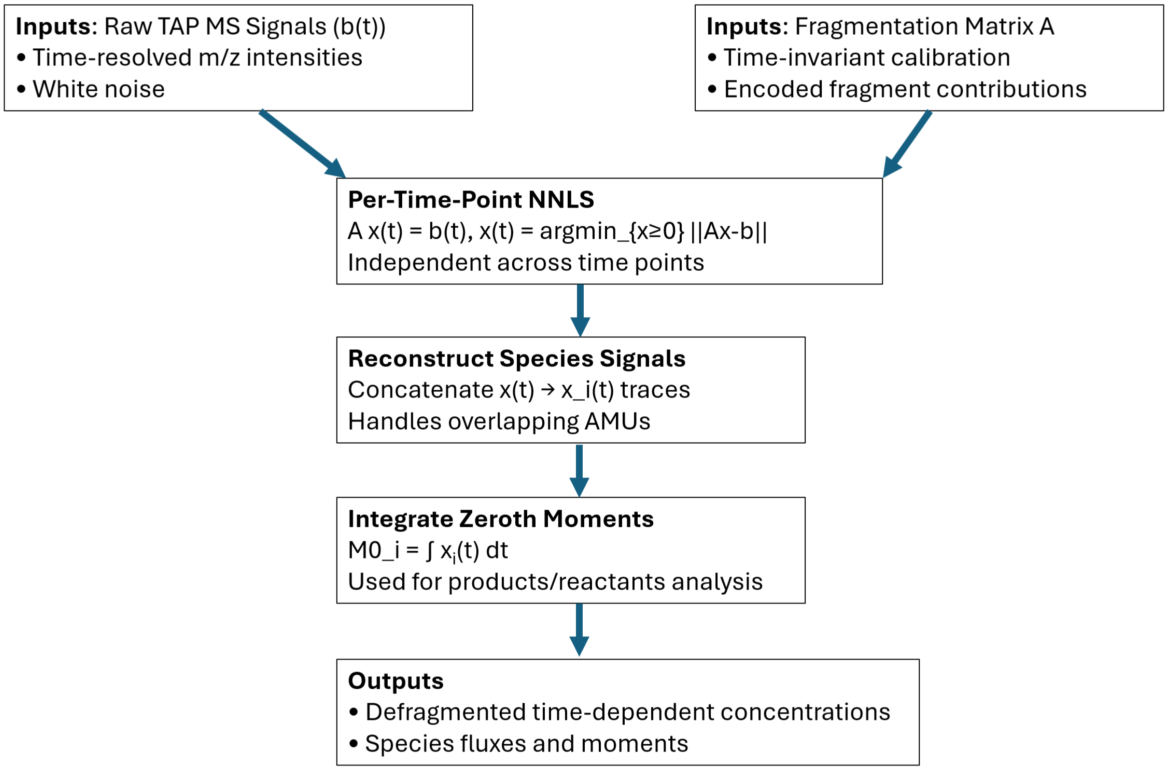

Scheme S1 - Flow diagram dictating order of operations and calculation steps as described in the "NNLS execution" section of this supporting information document.

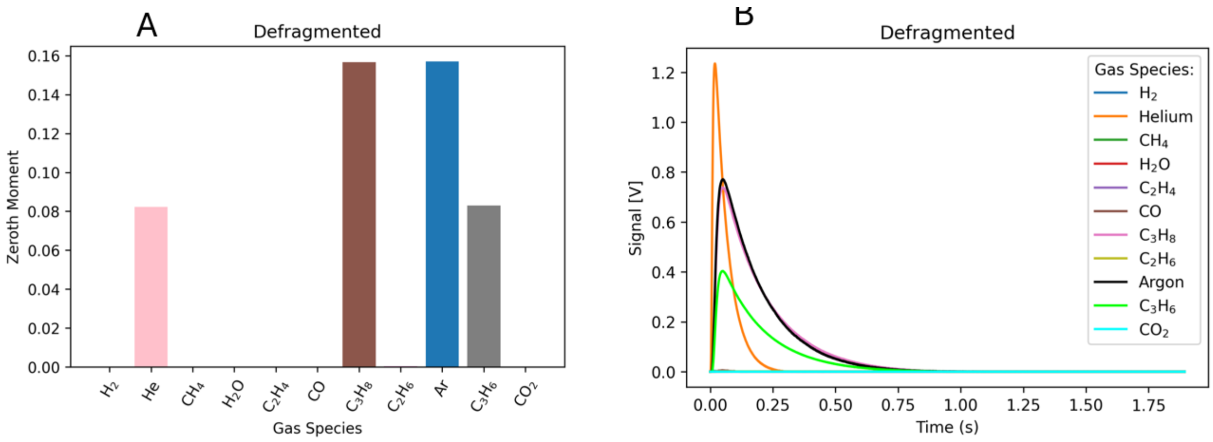

Figure S2 - (A) moment values for the inert pulsed during propane:argon and propylene:helium pulsing over a quartz-packed inert reactor, as a proof of concept for the defragmentation calculation. NOTE – the helium signal in (A) (zeroth moment value) is adjusted.

Figure S3 illustrates an example of how the NNLS defragments the MS signal at several single points in time based on the ten AMU values scanned during the PDH experiment. The top x-axis and blue data illustrate the ten-point signal-based fragmentation pattern at 0.25 seconds during one individual pulse cycle (cycle 3) during this co-pulsing experiment. The orange data and bottom x-axis illustrate what the species signal is at this same point in time following defragmentation, i.e., for a 6-second pulse, 6000+ of these calculation iterations are made to solve the fragmentation matrix problem. Figure S4 illustrates a mapping of the raw signals (matrix (b)) at these six points in time and the resultant defragmented data, clearly illustrating that the results of defragmenting these six points in time make up the two flux response curves from propane and propylene. Figure S6 displays a comparison of the propane and propylene co-pulsing experiment flux response curves (normalized) in comparison to propane and propylene curves from a control experiment where only one gas was pulsed at a time over the quartz-packed reactor. The results in this figure indicate that the co-pulsed curves represent the individually pulsed curves well and serve as further proof of the retention of flux curve properties following complex defragmentation.

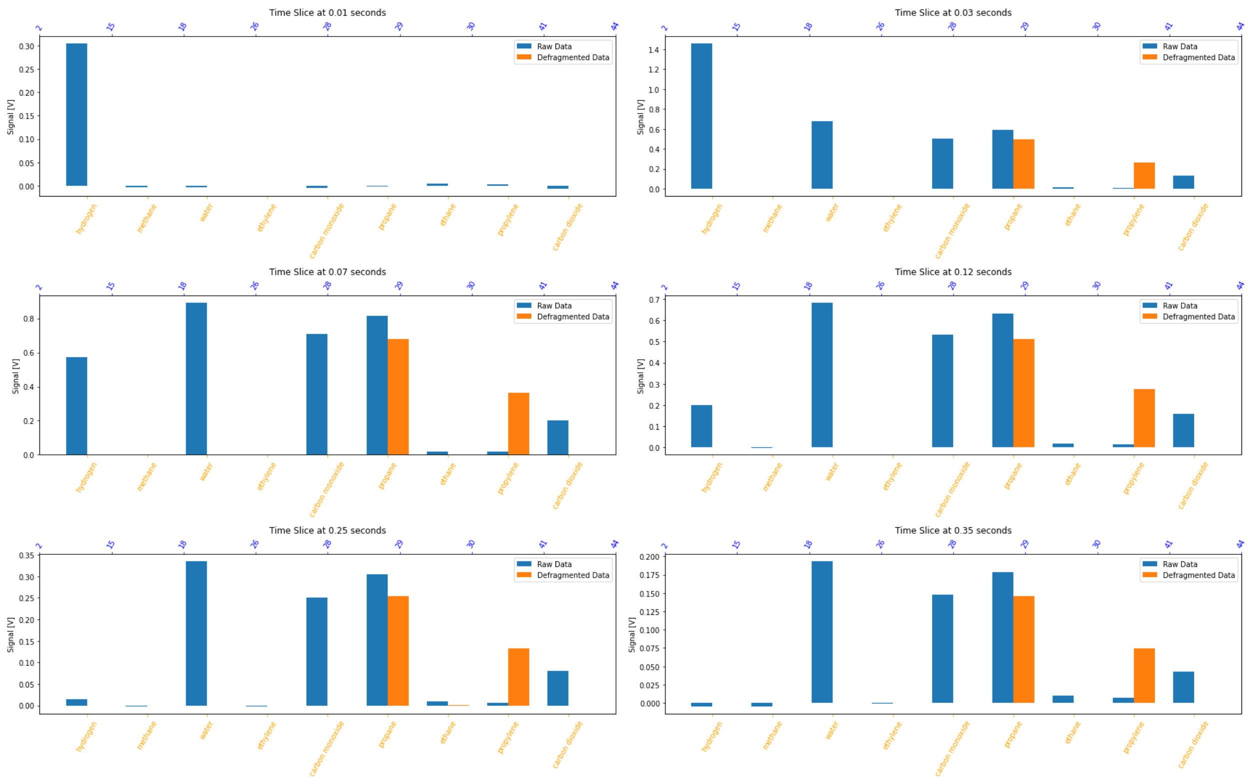

Figure S3 - the results of a single defragmentation iteration for the ten AMU species tracked during this experiment, at 0.10, 0.13, 0.18, 0.23, 0.35, and 0.45 seconds. Blue data indicates the fragmented raw data pattern at this time, and orange indicates defragmented.

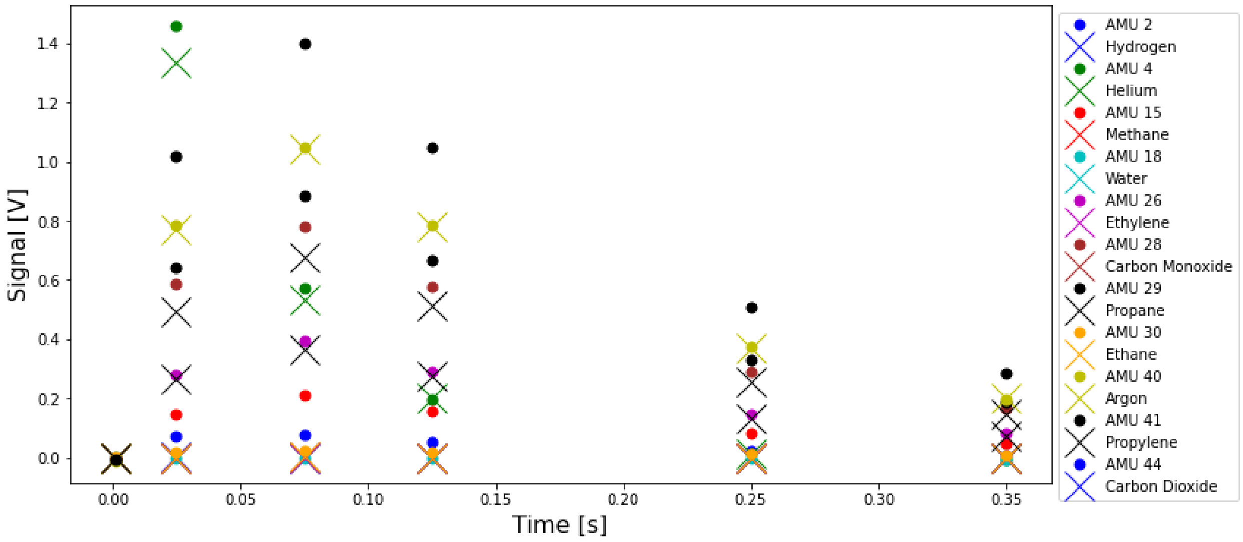

Figure S4 - Mapping of the raw defragmentation pattern (circle data) over the six time slices shown in Figure S4. The X data displays the defragmented signals for these slices, illustrating the reconstruction of the propane and propylene flux responses on a point-by-point basis.

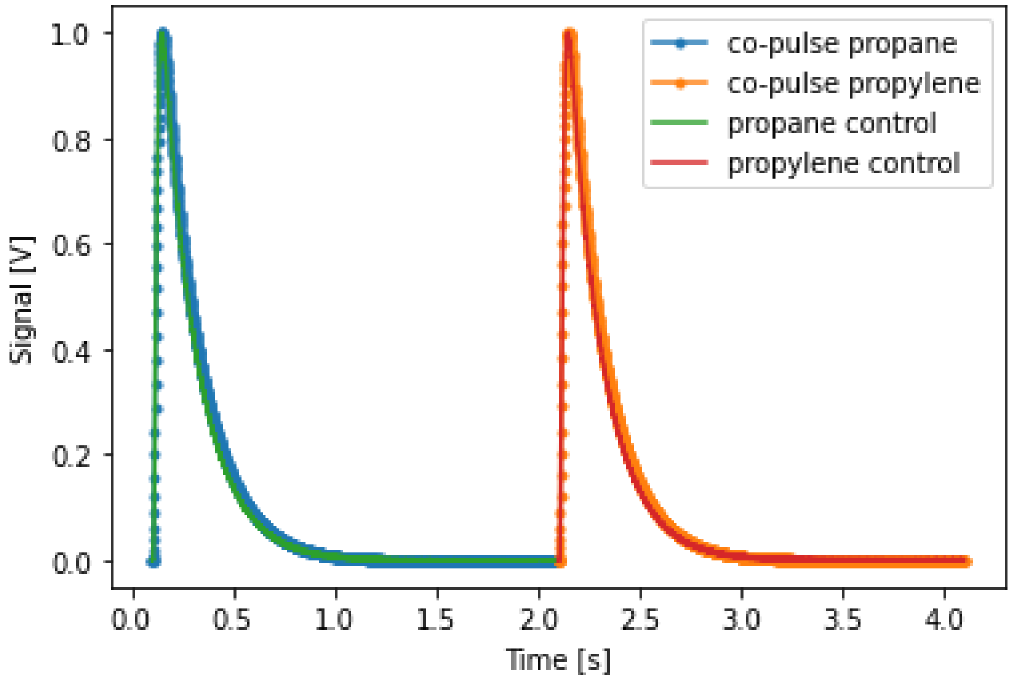

Figure S5 - Comparison of defragmented flux patterns from co-pulsing of propane and propylene in tandem over a quartz reactor with individual (control) pulses of propane and propylene (separate experiments) over the same quartz-packed reactor. Control data (only one species pulsed) is represented by the smooth lines, and co-pulsing data is represented with dots.

Calibration Drift with Time and Pulse Size

Collection of fragmentation calibration data and matrix construction should be performed often, as there are drifts in the MS signal over time/with usage. Figure S7 displays charts of how various fragmentation patterns change over the course of months for a single MS in the TAP reactor unit at Idaho National Laboratory. 8 AMU values are shown, and we observe large differences in moment value (Y axis). The data here indicates that a calibration is required at least monthly. Pulse size also affects the fragmentation matrix. Figure S8 displays the results of different pulse sizes on the fragmentation patterns. In this figure, the pulsing gas is the x-axis, and the Y-axis indicates how this changes with pulse size. The zoom-in features focus on Carbon Monoxide and Propane fragmentations and indicate there are no standard patterns/trends to correlate the fragmentation matrix with pulse size. This indicates that a separate fragmentation matrix should be collected for

all pulse sizes used in any given experiment. The reason for the lack of correlation of fragmentation with pulse size is difficult to pinpoint currently. The MS/TAP system is complex, and factors such as build up of materials on the TAP wall and build up of materials on the MS ionizer may affect the way gases are measured in the MS. This apparent "drift" in the MS systems could also contribute to the apparent error observed in defragmentation calculations that resulted in trace amounts of carbon species detection following defragmenting. After defragmenting the flux, zeroth moment values can be calculated as the area under the flux response curve for each AMU/species tracked, and interpretations can be made based on the qualitative and quantitative information stored in the MS response data.

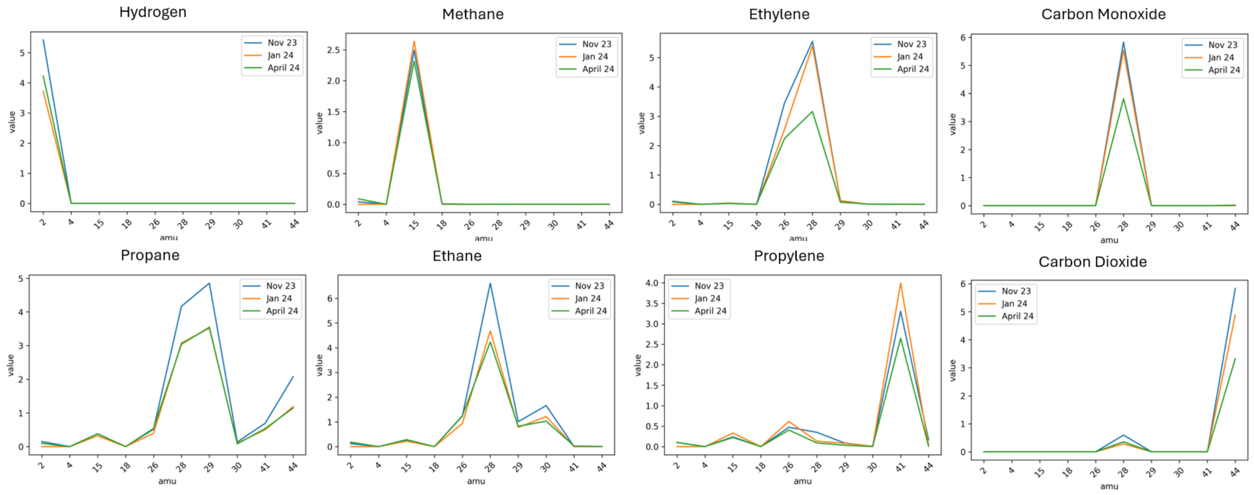

Figure S6 - Variation in fragmentation patterns for several pulsed gases over time. The colors in each graph represent when the data was collected (month and year). The different graphs represent different pulsed gases by the title, and the fragmentation pattern by the x-axis.

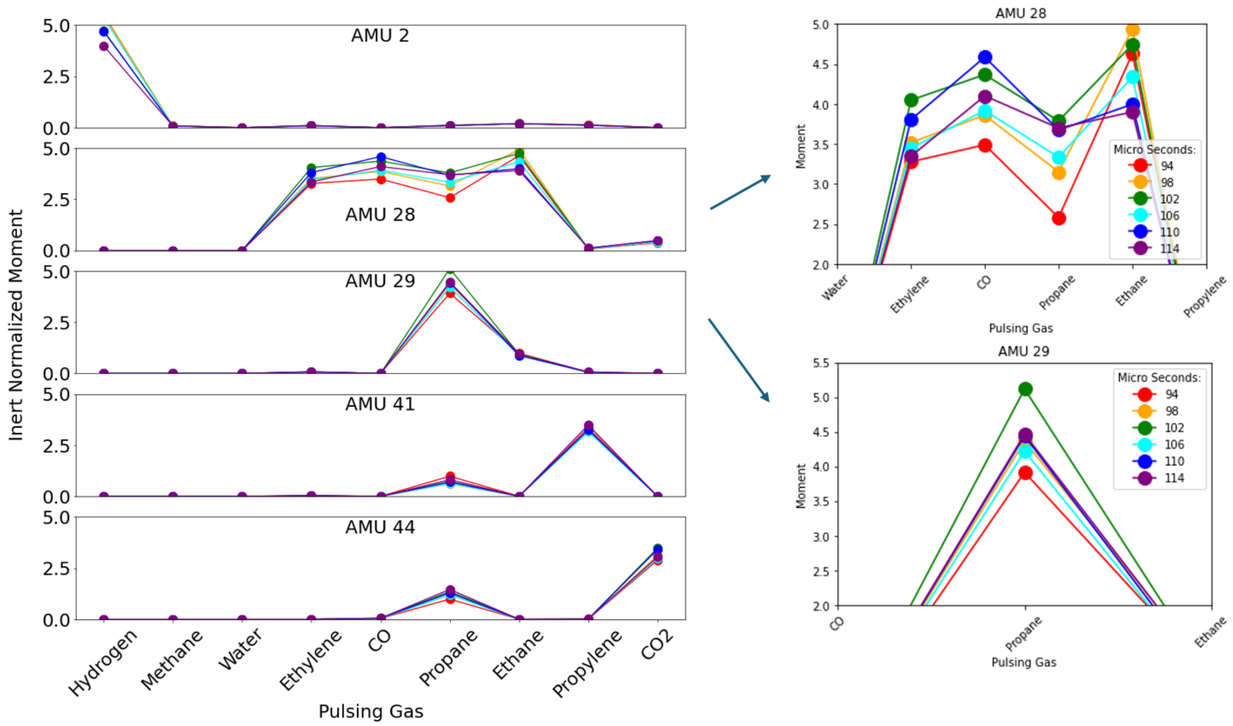

Figure S7- Fragmentation patterns for various pulsed gases with different pulse sizes. The pulse sizes (widths) are dictated by the different colors within each plot.

Rate and Concentration

The Y procedure utilizes a Laplace-domain analysis, which is then put through a Fourier transformation to model the rates and concentrations in the catalyst thin zone rather than the reactor exit (what is measured). The G procedure utilizes the Gamma distribution to extract rate and concentration at the catalyst zone without a predefined diffusion model. Unique time scalers are applied to either the flux (concentration) or the flux difference (rate) for the determination of R and C curves, which can be combined for the generation of an RC plot. The Y-procedure requires a few more processing steps, which have been discussed in detail elsewhere. [7,8] Following the calculation of the rate or concentration via either procedure, the curves must have their units converted properly. The details on how to calculate diffusion, as well as how to apply the amount

of moles in each pulse for conversion of each rate and concentration curve to nmol/s and mol/m^3, respectively, are covered in detail elsewhere [4].

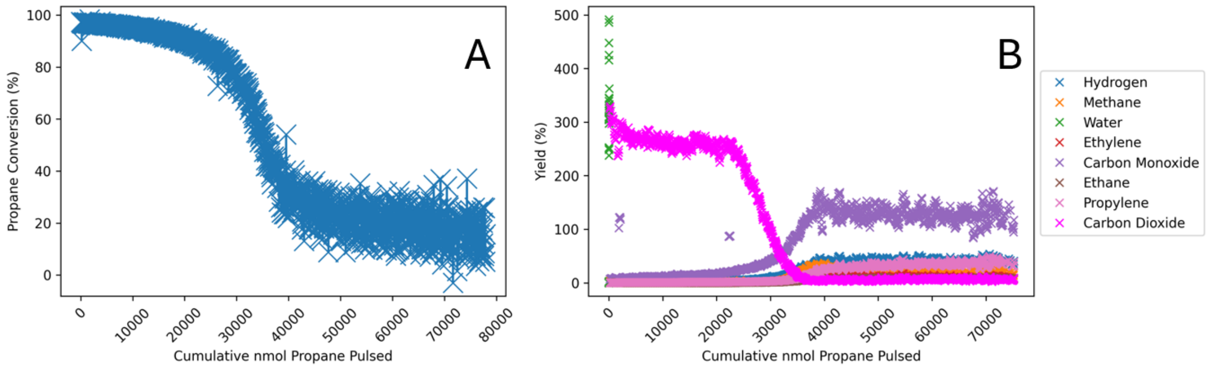

Figure S8 - Propane Conversion and Product Yield are A and B, respectively, acquired during continuous propane pulsing in TAP following O_2 pretreatment.

Conversion of Mass Spectrometer Signal to Moles

The unaveraged dataset is provided in Figure S9 and shows the same trend. Conversions and yields are acquired from the zeroth moment of each species following defragmentation of all signals over the course of the experiment. Yield is calculated for the averaged data as the ratio of moles of product formed to the number of converted moles of the reactant, propane. [9] The conversion is calculated with the difference in the moles of Argon and Propane. Recall that due to equal molar feeding, Argon contains an equivalent amount of moles as a propane pulse if no reaction occurs. It is useful to display the conversions and yields as the amount of nmol detected for each species over the course of the reaction (nmol), which is displayed for the same experiment in Figure 6. This is accomplished through a calibration to determine the amount of moles of total gas delivered in a single pulse (10 nmol per pulse here). Briefly, the amount of moles in a single pulse is determined from the subsequent pressure drop acquired from the release of pulses when the feed

to the pulse valve is blocked. The ratio of the measured product moment to the inert gas moment delivers the instantaneous nmol amount. The inset in Figure 6A zooms in on the Y-axis.

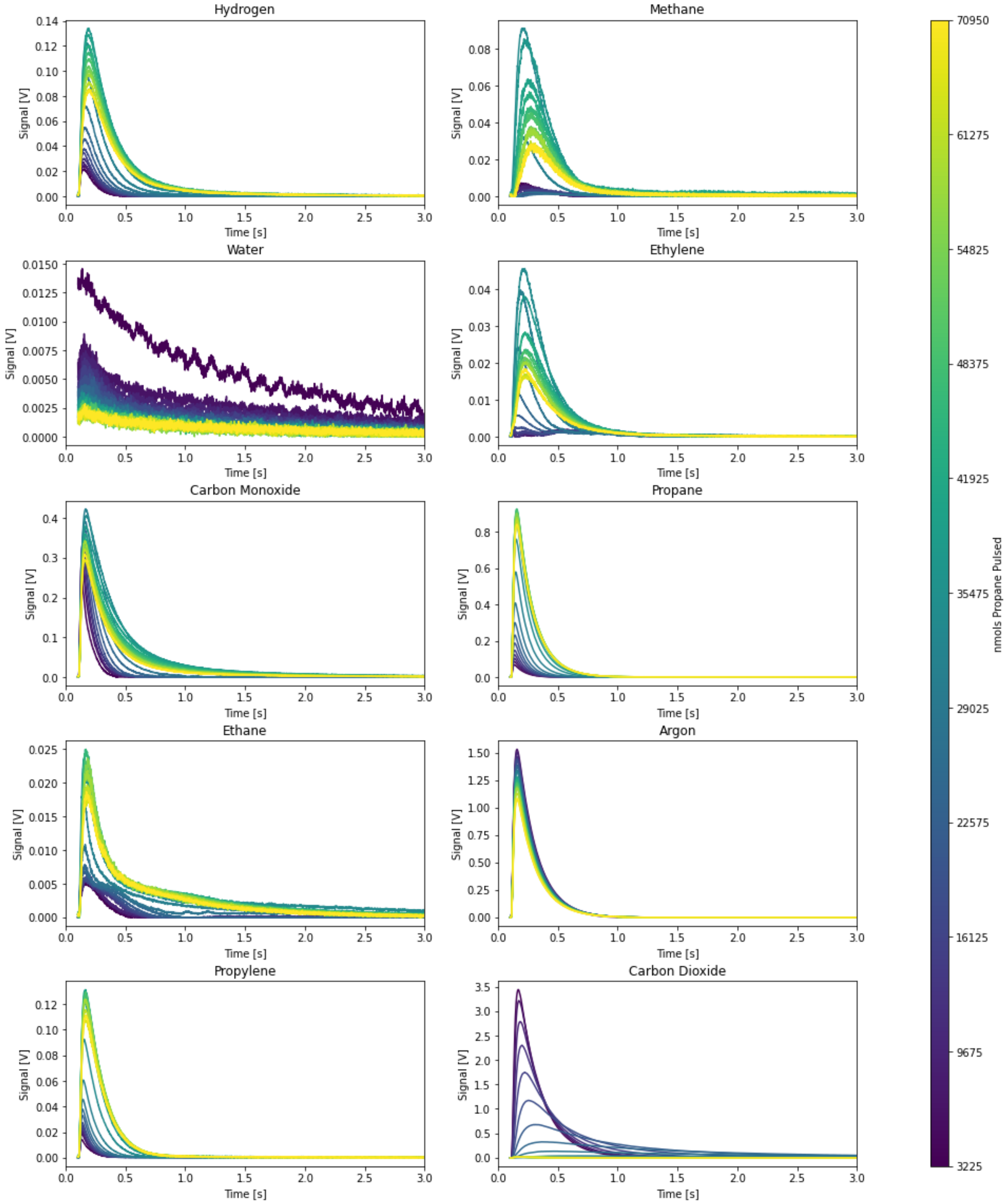

Figure S9 - Flux response signals for all detected species (omitting water) over the course of the propane pulsing in the Chrome catalyst following O_2 pretreatment.

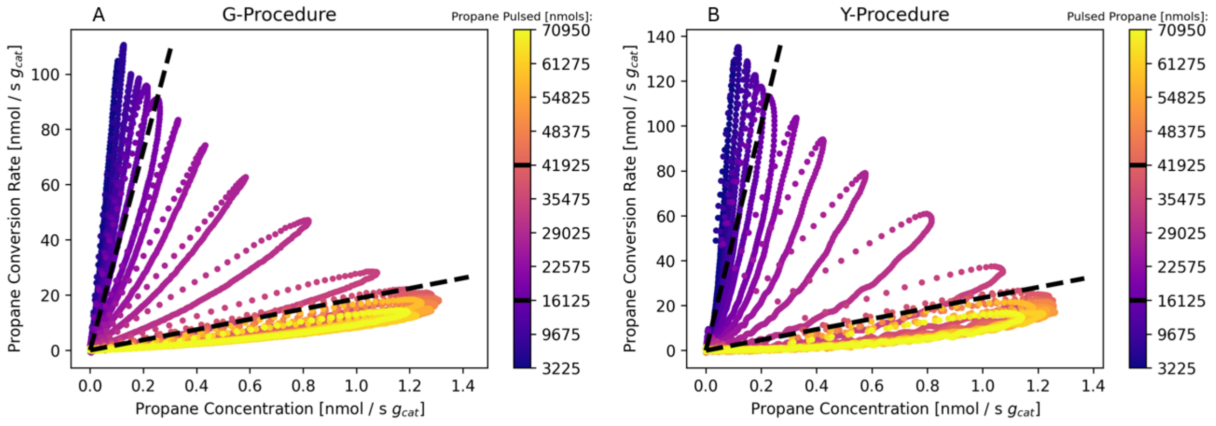

Figure S10 - Rate and Concentration petals calculated with either the G or Y procedure (6 pt smoothing) as labeled on each plot. The amount of nmol of propane pulsed following the color scheme purple to yellow. All y units = Rate [nmol/s g_{cat}]. All x units = Concentration [10^2 mol/m^3].

Figure S10 A and B differ only in their Rate/Concentration (RC) calculation methods: G and Y procedures, respectively. Both methods yield similar petal shapes and trends, indicating that the exit flux scaling method is less critical than defragmentation.

References:

1. *Benzenamine, N, N-dimethyl-*. 2025 [cited 2025; Available from: https://webbook.nist.gov/cgi/cbook.cgi?ID=C74986&Mask=200#Mass-Spec.
2. Knoll, D.A. and D.E. Keyes, *Jacobian-free Newton–Krylov methods: a survey of approaches and applications.* Journal of Computational Physics, 2004. **193**(2): p. 357-397.
3. Chen, D. and R.J. Plemmons, *Nonnegativity constraints in numerical analysis*, in *The birth of numerical analysis*. 2010, World Scientific. p. 109-139.
4. Kunz, M.R., et al., *Pulse response analysis using the Y-procedure: A data science approach.* Chemical Engineering Science, 2018. **192**: p. 46-60.
5. Wang, S., et al., *A Simulation Framework for Understanding Transport and Kinetics in Transient Reactor Experiments.* 2025.
6. Strang, G., *Introduction to linear algebra*. 2022: SIAM.
7. Redekop, E.A., et al., *The Y-Procedure methodology for the interpretation of transient kinetic data: Analysis of irreversible adsorption.* Chemical Engineering Science, 2011. **66**(24): p. 6441-6452.
8. Yablonsky, G.S., et al., *The Y-procedure: How to extract the chemical transformation rate from reaction–diffusion data with no assumption on the kinetic model.* Chemical Engineering Science, 2007. **62**(23): p. 6754-6767.
9. Fogler, H.S., *Elements of Chemical Reaction Engineering*. 5th ed. 2016, Pretince Hall.
